# Enhancing salinity tolerance in cultivated rice through introgression of African rice genes and application of moringa leaf extract

**DOI:** 10.1186/s12870-025-06102-y

**Published:** 2025-02-07

**Authors:** Heba A. Saleh, Shaimaa M. N. Tourky, Farag Ibraheem, Samy A. Abo-Hamed, Wafaa M. Shukry, Walid H. Elgamal, Eman M. Elghareeb

**Affiliations:** 1 Faculty of Science, Botany Department, Mansoura University, Mansoura, 35516 Egypt; 2 Biology and Chemistry Department, Umm Al-Qura University, Al-Qunfodah University College, Al-Qunfodah, 21912 Saudi Arabia; 3 Field Crops Research Institute (FCRI), Agricultural Research Center (ARC), Giza, Egypt

**Keywords:** Salinity, Rice, Moringa, Photosynthesis, Proline, Enzymatic antioxidants

## Abstract

**Background:**

Salinity is a major threat to rice growth and productivity. Utilizing wild rice-derived genes and biostimulants with high growth promoting- and stress-alleviating potential can significantly improve salinity tolerance in cultivated rice. Herein, we investigated the vegetative growth and physiological responses of Giza 177 (*Oryza sativa*, salinity sensitive, high-yielding cultivar) and a promising introgression salt tolerant line (*sativa/glaberrima*; SG 65) from a population of Giza 177 × African rice (*Oryza glaberrima*) under low (2.75 mS/cm) and high (5.5 mS/cm) salinity stress. The possible ameliorative effects of priming rice seeds in moringa leaf extract (MLE) on these responses were also tested.

**Results:**

The two salinity levels induced differential reduction in plant growth in both cultivars. In the MLE-unprimed plants, salinity induced 34–54% and 30–45% reductions in biomass accumulation in Giza 177 and SG 65, respectively. These responses were associated with significant differential reductions in relative water content, chlorophylls, carotenoids, and gas exchange parameters (transpiration rate, net photosynthetic rate, stomatal conductance, and intercellular CO_2_ concentration), ascorbic acid, and total protein. Conversely, salinity induced the accumulation of H_2_O_2_, malondialdehyde, proline, carbohydrate fractions, and membrane injury. MLE treatment mitigated the above salinity-induced adverse effects in both cultivars via reducing the salt-induced oxidative stress through the induction of non-enzymic (total phenols, and flavonoids) and enzymic antioxidants including ascorbate peroxidase, catalase, peroxidase, and polyphenol oxidase in both cultivars. SG 65 plants exhibited consistently higher salt tolerance and responsiveness to MLE than Giza 177.

**Conclusions:**

This study reports significant differences in an array of critical physiological and biochemical indices that underpin the divergent responses between the two salinized cultivars. It demonstrates the potential of African rice-derived genomic fragments and MLE priming in mitigating salinity stress, highlighting their use as a sustainable strategy for increasing rice production in salt-affected soils.

**Supplementary Information:**

The online version contains supplementary material available at 10.1186/s12870-025-06102-y.

## Introduction

Salinity is the second most prevalent abiotic stress after drought, causing unpredictable losses in global agriculture output [1]. It negatively impacts over 800 million hectares of agricultural land worldwide [2]. Approximately, 48 million hectares of land in South and Southeast Asia’s humid regions are technically suitable for rice cultivation, however, they remain unused or yield poor results because of salinity (https://www.fao.org/family-farming/detail/en/c/1618307/). Statistically, salinity causes detrimental economic losses in agriculture, estimated to be up to USD 27 billion annually [3]. Under the ongoing climatic change scenario and the continuously growing world population, the salinity-induced challenges will be more complicated, and the threat of salinity will increase in many irrigated lands [4]. Unfortunately, these problems will be more severe in developing countries due to water shortages, improper irrigation, poor drainage practices, and low soil fertility [5]. Therefore, adopting novel strategies to enhance salt tolerance in cultivated cereals which are severely sensitive to salinity, is critical to sustain the world food supply.

Rice (*Oryza sativa* L.) is one of the most important economic cereals, providing food and energy for more than 50% of the world’s population, with global production reaching 512.6 million tons in 2022 [6, 7]. Rice belongs to the family *Poaceae* and is a member of the genus *Oryza* which has many species with only two cultivated species: *O. sativa*, predominantly grown in Asia, and *O. glaberrima*, native to West and Central Africa [8]. Salt tolerance is a complex trait and is greatly affected by the environment, with significant genetic variability among rice species and cultivars [9]. Rice is currently listed as the most salt-sensitive cereal, with a threshold of 3 dSm^− 1^ for most cultivated varieties [10]. The responses of rice plants to salinity are shaped by several factors including the cultivar, growth stage, plant organs, and stress magnitude [11].

Salinity stress retards rice growth and productivity through various mechanisms. It increases the cellular levels of Na^+^ and Cl^−^ ions, causing ionic stress that reduces water and essential elements uptake, thereby diminishing overall plant growth [12]. The resultant ionic imbalance decreases photosynthetic pigments, affecting sugar metabolism. Long-term salinity exposure reduces gas exchange, hinders photosynthesis and CO_2_ assimilation, and disrupts carbon and nitrogen metabolism [13]. Salinity also disrupts the K^+^ to Na^+^ ratio, causing osmotic imbalance and the production of reactive oxygen species (ROS), leading to altered cell turgidity, loss of membrane structure and integrity, and ultimately cell death [14].

To cope with salinity-induced damage, rice plants enhance enzymatic and non-enzymatic antioxidant machinery to detoxify ROS and consequently minimize oxidative stress [15]. Further, they induce the synthesis of osmolytes such as proline and ascorbate to boost salt resistance [16]. Interestingly, numerous studies have shown significant genetic variability in salinity tolerance among rice cultivars and suggested that tolerant genotypes may employ different response mechanisms [17]. Agricultural organizations worldwide have emphasized the importance of genetic diversity along with improved crop management, enhanced selection for salt tolerance, and a better understanding of salt tolerance mechanisms for production of high-yielding rice genotypes in salt-affected lands [11, 18]. These efforts will aid breeding programs in meeting the growing global demand for rice.

Along with developing salt-tolerant genotypes, various biostimulants with high growth-promoting and stress-alleviating potential can significantly enhance salt tolerance in cultivated rice. Moringa leaf extract (MLE) has gained widespread recognition as a safe biostimulant in agricultural applications in normal and stressful environments [19]. It is cost-effective, easy to prepare, and environmentally friendly [20]. MLE contains a diverse array of vitamins, ascorbate, essential minerals, hormones, and amino acids, which promote several physiological and biochemical processes in plants. It improves fresh and dry masses, chlorophyll contents, photosynthetic rate, stomatal conductance, ascorbic acid, total soluble proteins, antioxidant enzymes, and phytohormones in rice under water deficit conditions [21]. Many reports have investigated the potential of MLE in ameliorating salinity stress in various crops including cotton [22], wheat [23], and canola [24]. However, the mechanisms underlying MLE’s ameliorative effects of salinity stress in rice are not fully understood.

Giza 177 (*O. sativa)* is an Egyptian rice cultivar known for its early maturity, high yield, and excellent cooking and eating features, but its growth and productivity are greatly diminished in salt-affected lands [25]. African rice (*O. glaberrima*), originating from its wild relative *O. barthii*, is well adapted to African conditions and exhibits considerable natural tolerance against severe abiotic stress including salinity but generally has lower yield potential than *O. sativa* [26–28]. *O. glaberrima* is a rich source of genes for tolerance to various biotic and abiotic stresses [29] and thus can provide a variety of agronomically important traits for rice improvement. In the current study, we hypothesize that introgression of African rice genes into Giza 177 genetic background can significantly improve its growth and physiological responses under normal and salinity stress conditions. In addition, these responses can be further improved via priming rice seeds in MLE. Therefore, we simultaneously investigated the vegetative growth and its underlying physiological responses of Giza 177 and a promising salt tolerant line (*Sativa*/*glaberrima*; SG 65) derived from Giza 177 × African rice (*O. glaberrima*) a population under two salinity levels: low (2.75 mS/cm) and high (5.5 mS/cm) with and without MLE priming. This study will give insights into the potential of combining African rice genes and MLE for the sustainable enhancement of salt tolerance in rice.

## Materials and methods

### Genetic stocks

Two rice cultivars: Giza 177 [Pedigree: Giza 171/Yomji No1) Pi No.4] and a promising salt tolerant line (*Sativa*/*glaberrima*; SG 65) derived from Giza 177 × African rice (*O. glaberrima*, IRGC 101901) population were used in the current study. The seeds of both cultivars were obtained from the Rice Research and Training Center, Sakha, Kafr El-Sheik, Egypt.

## MLE preparation and analysis

Mature, fresh, healthy, and disease-free leaves were collected from six-year-old moringa trees that were already established at the experimental nursery of the Faculty of Agriculture, Mansoura University, Mansoura (31° 2’ 16.5588’’ N and 31° 22’ 53.4828’’ E), Egypt. The collected leaves (1 kg) were rinsed with tap water, and ground in 100 mL of distilled water using an electric grinder for 15 min. The water-soluble fraction was collected and centrifuged at 8000 g for 15 min at 4 ˚C. The obtained MLE was diluted using distilled water at a ratio of 1:30 (v/v) immediately before application, according to [30]. The bioactive components of MLE were analyzed and presented in Supplementary Table 1.

## Experimental setup, sowing, maintenance, transplanting, and recovery

The experiments were carried out during rice cultivation season in 2023 at the Department of Botany, Faculty of Science, Mansoura University, Egypt, under natural growing conditions. The average day/night temperatures were 39.2 ± 4 ^°^C and 23.5 ± 5 ^◦^C and the relative humidity was 55%. Uniform and healthy seeds of Giza 177 and SG 65 cultivars were surface sterilized in 3.6% NaClO for 15 min and rinsed thrice with distilled water. Seeds were then divided into two groups: the first group was primed in aerated distilled water as a control, whereas the second group was primed in aerated MLE. The two groups were incubated in the dark for 8 h at 27 °C. The ratio of seed weight to priming solution was 1:5 (w/v) [30]. Each of the control and MLE-treated seeds of the two cultivars were sown in 10 plastic pots (30 cm in diameter and 25 cm in depth) filled with 7 kg of soil (clay: sand; 2:1, the chemical composition is shown in Supplementary Table 2). Pots were kept flooded with tap water (0.55 mS/cm) in a greenhouse for 28 days (nursery stage). After 28 days, healthy and uniform plantlets from each cultivar were transplanted into bigger pots (30 cm in diameter, 40 cm in depth, containing 10 kg of the same soil, 10 plantlets/pot, 18 pots for each cultivar). Plantlets were maintained in the greenhouse (12 h light/ 12 h dark cycle and 450 µmol m^− 2^ s^− 1^ light intensity) until complete recovery and establishment. The recovered plants were thinned to 5 plants/pot and used for salinity stress application.

## Salinity stress application, maintenance, and sample collection

Two levels of salinity stress were prepared: S_1_ (low stress, 2.75 mS/cm, pH 8.29) and S_2_ (high stress, 5.50 mS/cm, pH 8.31). S_1_ and S_2_ were prepared by diluting stock seawater (54.6 mS/cm, pH 10.5) with fresh tap water (0.55 mS/cm, pH 8.1) to achieve the required EC values. The stock seawater was obtained from the Gamasa region, Mediterranean Sea, (31°29 ‘16.1"N and 31°32’03.3"E) and has a pH 10.5, cations (Na^+^ 7400, K^+^ 340, Ca^2+^ 519, Mg^2+^ 1395 ppm), and anions (HCO_3_^−^ 91, Cl^−^ 1786 ppm). The EC of all solutions was monitored using a portable electrical conductivity meter (HANNA Instrument, HI 8033). The experiment was conducted in a completely randomized design (three biological replicates/ treatment), with the factorial arrangement primarily due to three factors: Factor 1: Rice cultivars (Giza 177 and SG 65), Factor 2: MLE treatment, and Factor 3: salinity treatments. Pots with homogenous control and MLE-treated plants from each cultivar were allocated into three subgroups. The control and MLE-treated plants were flooded with equal volumes of either tap water, 2.75 mS/cm, or 5.50 mS/cm every 4 days. A summary of the treatments is shown in Table 1.

**Table 1:** The treatments applied in the current study

Treatments		
**Seeds primed in**	**Plants irrigated with**	**Code**
Water	Water	Control (C)
	2.75 mS/cm	S_1_
	5.50 mS/cm	S_2_
MLE	Water	MLE
	2.75 mS/cm	MLE + S_1_
	5.50 mS/cm	MLE + S_2_

Plants were maintained in the greenhouse until 56 days after sowing. At this point, the entire plants were uprooted, thoroughly washed with distilled water, separated into roots and shoots, and prepared for downstream analysis. One set of fresh plant samples was immediately submerged in liquid nitrogen, stored at − 80 °C, and used for photosynthetic pigments, malondialdehyde (MDA), protein content, proline, and enzyme analysis. Another set of fresh plant samples was used for growth trait measurements, oven-dried to a constant weight at 70 °C, and used for biochemical and elemental analyses. All biochemical analyses were carried out on three biological replicates.

## Growth analysis

The root and shoot lengths of ten plants were measured from the root-shoot junction to the tip of the longest root and tip of the innermost leaf within the whorl, respectively. Fresh and dry weights were recorded using a digital balance. Flag leaf area was calculated using the equation (Leaf area = length × breadth × 0.75) [31]. Other root characteristics were calculated including root distribution (fresh mass/length), root density (dry mass/length), and root/shoot ratio. Also, root volume was determined by using the water displacement method [32].

### Relative water content (RWC)

Uniform disks from three fresh flag leaf discs from each treatment were initially weighed (FWT) and then immersed in distilled water for 4 hours to determine the turgid weight (TWT). Subsequently, leaves were dried in an electric oven at 70 °C for 24 hours and weighed (DWT). RWC was estimated as (FWT - DWT)/ (TWT - DWT) × 100, according to [33].

## Determination of K^+^ and Na^+^ concentrations

Known weights of the dried and powdered shoot and root samples were digested in a mixture of 5 mL nitric acid and 1 mL perchloric acid as described previously [34]. The Na^+^ and K^+^ contents were measured using a flame photometer (PFP7, Jenway), and the results were reported in mmol g^− 1^ DWT.

## Determination of photosynthetic pigments and gas exchange characteristics

A known weight of a fully expanded flag leaf was extracted with dimethyl sulfoxide (DMSO) as described in [35] and the absorbance of extracts was measured at 470, 645, and 663 nm by using a Shimadzu UV-160 A spectrophotometer. Concentrations were calculated and expressed as mg g^− 1^ FWT [36]. Gas exchange parameters (transpiration rate (*E*), net photosynthetic rate (*P*_*N*_), stomatal conductance (*g*_*s*_), and intercellular CO_2_ concentration (*C*_*i*_)) were measured in the fully expanded flag leaf using a portable photosynthesis system LCi-SD (Analytical Development Company, Hertfordshire, UK) on a clear sunny day between 10 AM and 12 AM as described previously [37]. The leaf water use efficiency (WUE) was computed as *P*_*N*_*/E* [38].

### Estimation of carbohydrate fractions

Known weights of the dried flag leaves were extracted in 80% (v/v) ethanol at 25 °C according to [39]. The ethanolic extracts were used for the spectrophotometric determination of sucrose at 620 nm [40] and total soluble sugars (TSS) at 625 nm using anthrone reagent [40]. Total carbohydrates (TC) were extracted by mixing 0.1 g of dry tissue with 5 mL of HCl (2.5 N) in a boiling water bath for 3 h, then neutralizing with Na_2_CO_3_ after cooling to ambient temperature. The TC in the extract was determined spectrophotometrically using anthrone reagent at 630 nm [41]. All carbohydrate fractions were expressed as mg g^− 1^ DWT.

### Estimation of total soluble proteins (TSP), proline, and ascorbic acid

A known weight of the frozen flag leaf was extracted in Tris-HCl buffer (pH 8, 0.2 M) as described previously [42]. TSP in the extracts was determined spectrophotometrically at 595 nm using the dye-binding assay with Coomassie Brilliant Blue G250 [43]. The concentration of TSP was calculated using a bovine serum albumin standard curve and expressed as mg g^− 1^ FWT.

A Known weight of frozen flag leaf was extracted in 3% sulfosalicylic acid and the homogenate was filtered through Whatman No.1. Two mL of glacial acetic acid were added to 2 mL of the filtrate and left in a boiling water bath. After an hour, the mixture was incubated in an ice bath to terminate the reaction. The reaction mixture was mixed strongly with 4 mL toluene using an agitator for 15–20 s. The chromophore containing toluene was separated from the aqueous phase, warmed to room temperature, and the absorbance was measured at 520 nm using toluene as blank [44]. Proline concentration was measured using a standard curve and expressed as mg g^− 1^ FWT.

The level of ascorbic acid (AsA) was measured as described in [45]. About 0.5 mL of the supernatant was mixed with 0.1 mL of the 2,4 dinitrophenylhydrazine/thiourea/copper (DTC) solution, followed by a 3 h incubation at 37 °C. Then, 0.75 mL of ice-cold, 65% sulfuric acid was added, and the resultant solutions were allowed to stand at room temperature for an additional 30 min before recording the absorbance at 520 nm.

### Assessing the oxidative stress damages

Electrolyte leakage (EL) in fully developed fresh flag leaves was evaluated following the procedure of [46] using an EC meter (HANNA Instrument, HI 8033). Leaves were cut into uniform pieces, placed into test tubes containing 30 mL of deionized water, incubated in the dark at room temperature for 24 h and the initial electrolyte conductivity (EC_1_) was recorded. The tubes were then incubated in a boiling water bath at 95 °C for 20 min, cooled to room temperature, and the final electrical conductivity (EC_2_) was recorded. The EL was calculated using the equation [EL (%) = EC1/EC2 × 100].

Hydrogen peroxide (H_2_O_2_) and malondialdehyde (MDA) were extracted by homogenizing 0.5 g of frozen flag leaf tissues in chilled trichloroacetic acid (TCA, 0.1%) at 4 °C. The homogenate was centrifuged at 12,000 rpm for 10 min at 4 °C. The supernatant was gathered and used for spectrophotometric determination of MDA and H_2_O_2_. MDA was measured using the Thiobarbituric acid method [47]. One mL of the plant extract was mixed with 4 mL of TCA (20%) containing TBA (0.5%), incubated in a water bath at 90 ˚C for 30 min, and cooled to room temperature. The mixture was centrifuged for 10 min at 10,000 rpm at 4 °C and the absorbance of the developed color was determined at 532 and 600 nm using a spectrophotometer. The absorbance at 600 nm was deducted from the absorbance at 532 nm and MDA concentration was calculated using the extension coefficient of 155 × 10^− 3^µM^− 1^ cm^− 1^ and then expressed as µmol g^− 1^ FWT.

H_2_O_2_ content was determined according to [48]. A 0.5 mL aliquot of leaf extract was thoroughly mixed with 0.5 mL of phosphate buffer (100 mM, pH 7.0) and 2 mL of KI (1 M). The mixture was incubated in the dark for 1 h and the absorbance was measured at 390 nm. The H_2_O_2_ content was determined using a H_2_O_2_ standard curve and expressed as µmol g^− 1^ FWT.

### Efficiency of the antioxidant system

#### Non-enzymic antioxidant compounds

Total phenols and flavonoids were extracted following the procedure outlined by [49]. For total phenolics, 50 µL of the methanolic leaf extract was combined with 400 µL Folin–Ciocalteu reagent and incubated at 25 ^◦^C for 3 min. Subsequently, 800 µL of sodium carbonate (10%) was added, and the tubes were vortexed and kept at 25 ^◦^C for 2 hours in the dark. The absorbance was recorded by spectrophotometry (Shimadzu model UV-160 A) at 765 nm and expressed as mg gallic acid equivalent (GAE) g^− 1^ DWT [50]. To assess total flavonoid content, aliquots (1 mL) of the methanolic extracts were mixed with 4 mL of distilled H_2_O and a 0.3 mL NaNO_2_ solution (5%). Then 0.3 mL of 10% AlCl_3_ was added to the tubes after 5 min and incubated at 25 ^◦^C for 6 min. Then, 2 mL of 1 N NaOH was added, followed by 2.4 mL of distilled water, and the reaction mixture was well mixed. The absorbance of the formed color was recorded at 510 nm and expressed as mg quercetin equivalent g^− 1^ DWT [51].

### Antioxidant enzyme activities

Antioxidant enzymes, including catalase (CAT), peroxidase (POD), polyphenol oxidase (PPO), and ascorbate peroxidase (APX) were determined in the frozen flag leaf samples as described previously [52]. Known weights were homogenized in cold phosphate buffer (0.02 M, pH 7) followed by centrifugation at 10,000 rpm for 20 min at 4 °C. The resulting supernatants were collected and used as enzyme extracts.

CAT activity was monitored according to [53]. Aliquots of 0.5 mL enzyme extracts were mixed with 1 mL phosphate buffer (0.01 M, pH 7.0), 0.5 mL of H_2_O_2_ (0.2 M), and 0.4 mL of distilled H_2_O. After 1 min, enzymic reactions were terminated by adding 2 mL of the acid reagent (dichromate/acetic acid mixture; 1/3 v/v). The developed color was measured at 610 nm using a spectrophotometer (Shimadzu model UV-160 A) and CAT activity was measured using H_2_O_2_ standard curve and expressed as mM H_2_O_2_ consumed min^–1^ g^–1^ FWT.

POD and PPO activities were assayed spectrophotometrically at 420 nm following the procedures described by [54]. For POD, the reaction mixture contained 0.1 mL of enzyme extract, 3 mL of pyrogallol (0.05 M) prepared in phosphate buffer (0.1 M, pH 6), and 0.5 mL of H_2_O_2_ (1%). The mixture was incubated for 1 min at 25 °C and the enzymatic reaction was stopped by 1 mL of H_2_SO_4_ (2.5 N). POD enzyme unit was defined as a unit per min^–1^ g^–1^ FWT. PPO was measured by mixing 1 mL of enzyme extract, 1 mL of pyrogallol (0.1 M), and 2 mL of phosphate buffer (0.02 M, pH 7) for 1 min at 25 °C. Then, 1 mL of H_2_SO_4_ (2.5 N) was added to stop the enzymatic reaction. PPO enzyme unit was defined as unit min^–1^ g^–1^ FWT.

APX activity was assessed following the method of [55]. The reaction mixture contained 50 mM potassium buffer (pH 7.0), 0.5 mM AsA, 0.1 mM H_2_O_2_, 0.1 mM EDTA, and 0.05 mL enzyme extract in a final volume of 700 µl. The activity was measured by recording the decrease in the absorbance at 290 nm for 1 min using an extinction coefficient of 2.8 mM^− 1^ cm^− 1^.

### Statistical analysis

All data were expressed as the means of three biological replicates ± standard error (SE). The data was statistically analyzed using a three-way analysis of variance (ANOVA) using CoStat Version 6.3 software to determine the impact of cultivars, MLE, and salinity treatment. GraphPad Prism 9.0.2 (GraphPad Software, Inc., La Jolla, CA, USA) was used to design the figures. The means of the tested treatments were compared using Post Hoc Duncan’s multiple range test at *p* ≤ 0.05. The principal component analysis (PCA) was performed with Origin Pro 9.8.0.200 software and Pearson’s correlation heatmap in GraphPad Prism.

### Experimental results

#### Effect of MLE on shoot traits of salinized rice cultivars

Salt treatments significantly reduced all growth indices including shoot length, fresh and dry weight, number of tillers, leaf area, and RWC in both cultivars compared to control plants (*p* ≤ 0.05) (Table 2). The reductions were more pronounced in Giza 177 than in SG 65. Under high salinity (S_2_), Giza 177 showed reductions of 58% in shoot fresh weight, 54% in dry weight, 60% in number of tillers, 25% in leaf area, and 11% in RWC, while SG 65 showed reductions of 49%, 44%, 50%, 21%, and 9%, respectively. S_1_ also caused significant but less severe reductions. MLE priming significantly increased (*p* ≤ 0.05) all the shoot parameters and RWC of both cultivars when compared to unprimed plants (Table 2). MLE-induced increases in shoot dry weight and number of tillers were particularly notable, with increases of 39% and 30% in Giza 177 and 36% and 40% in SG 65, respectively. Under salinity stress, MLE significantly mitigated the salinity-induced reductions in growth indices. MLE increased shoot dry weight and number of tillers by 28.7% and 50% in Giza 177 and by 29.7% and 57.1% in SG 65 under low salinity. The corresponding MLE-induced increases under high salinity were 41.2% and 37.5% in Giza 177, and 22.6% and 70% in SG 65. The interaction between salinity stress and cultivars was non-significant (*p* ≤ 0.05).

**Table 2:** Effect of MLE on shoot growth parameters and RWC of seawater-stressed rice cultivars at the vegetative stage

Varieties	Treatments	Shoot length (cm)	Shoot FWT (g)	Shoot DWT (g)	Tillers number /plant	Flag Leaf area (cm^− 2^)	RWC (%)
**Giza 177**	C S_1_ S_2_ MLE MLE + S_1_ MLE + S_2_	65.60 ± 0.16b 62.72 ± 0.27c 59.75 ± 0.25d 68.50 ± 0.426a 64.55 ± 0.33b 62.50 ± 0.14 c	10.51 ± 0.14b 6.04 ± 0.17ef 4.36 ± 0.10 g 12.79 ± 0.27a 7.57 ± 0.07 cd 6.16 ± 0.20ef	2.76 ± 0.08d 1.82 ± 0.03 g 1.26 ± 0.07 h 3.83 ± 0.04b 2.35 ± 0.08e 1.78 ± 0.07 g	5.00 ± 0.00bc 3.00 ± 0.00ef 2.00 ± 0.00 g 6.500 ± 0.29a 4.50 ± 0.23c 2.75 ± 0.25efg	30.35 ± 0.96b 24.93 ± 0.30cde 22.54 ± 0.28f 32.38 ± 0.19a 28.52 ± 0.63b 25.36 ± 0.62 cd	61.63 ± 0.88d 59.31 ± 0.35e 54.68 ± 0.31 g 64.41 ± 0.20b 61.43 ± 0.46d 57.18 ± 0.46f
**SG 65**	C S_1_ S_2_ MLE MLE + S_1_ MLE + S_2_	58.12 ± 0.82e 52.70 ± 0.58 g 47.30 ± 0.75i 60.62 ± 0.90d 56.12 ± 0.92f 50.82 ± 0.33 h	11.05 ± 0.49b 6.97 ± 0.47de 5.57 ± 0.33f 13.69 ± 0.67a 8.27 ± 0.32c 6.74 ± 0.29de	3.03 ± 0.06c 2.11 ± 0.02f 1.67 ± 0.07 g 4.11 ± 0.03a 2.73 ± 0.04d 2.05 ± 0.08f	5.00 ± 0.41bc 3.50 ± 0.29de 2.50 ± 0.29 fg 7.00 ± 0.41a 5.50 ± 0.29b 4.25 ± 0.48 cd	26.42 ± 0.82c 24.08 ± 0.76def 20.63 ± 0.75 g 28.96 ± 0.88b 25.57 ± 0.87ef 23.00 ± 0.12ef	63.74 ± 0.66b 61.79 ± 0.51 cd 57.53 ± 0.45f 66.70 ± 0.29a 63.04 ± 0.18bc 59.88 ± 0.20e
**Varieties (V)**		***	ns	ns	**	***	ns
**Moringa Leaf Extract (MLE)**		*	ns	ns	ns	*	ns
**Salinity (S)**		***	ns	ns	ns	*	ns
**V × MLE**		***	*	**	***	**	***
**V × S**		***	ns	*	**	***	ns
**MLE × S**		*	**	**	***	***	**
**V× MLE × S**		***	***	***	***	***	***

Values are means ± SE of three replicates. Different letters indicate significant responses whereas those followed by the same letters depict non-significant responses for the respective parameters at Post Hoc Duncan’s multiple range test (*p* ≤ 0.05). C; Control, S_1_; salinity level 1, S_2_; salinity level 2, MLE; moringa leaf extract, FWT; fresh weight, DWT; dry weight. *, **, and *** indicate significant differences at *p* ≤ 0.05, 0.01, and 0.001and ns indicates non-significant differences

### Effect of MLE on root traits of salinized rice cultivars

Salinity negatively affected root characteristics in both cultivars (Table 3). Under high salinity, Giza 177 showed significant reductions (*p* ≤ 0.05) in root length (30%), root/shoot ratio (22.7%), root fresh weight (56%), root dry weight (60%), root volume (52%), root distribution (38%), and root density (43%) compared to control plants. SG 65 showed reductions of 28%, 11.4%, 43%, 42%, 59.6%, 21%, and 19%, respectively. MLE treatment under both salinity levels improved all root traits compared to stressed, unprimed plants. The highest MLE-induced increases under S₂ were observed in root volume and root dry weight, with increases of 39.5% and 47.2% in Giza 177, and 68% and 21.5% in SG 65, respectively. The interaction between salinity stress and cultivars was non-significant (*p* ≤ 0.05).

**Table 3:** Effect of MLE on root growth parameters of seawater-stressed rice cultivars at the vegetative stage

Varieties	Treatments	Root length (cm)	Root / Shoot ratio	Root FWT (g)	Root DWT (g)	Root volume (cm^3^)	Root distribution (g cm^− 1^)	Root density (g cm^− 1^)
**Giza 177**	C S_1_ S_2_ MLE MLE + S_1_ MLE + S_2_	20.77 ± 0.37c 18.32 ± 0.45d 14.62 ± 0.24f 23.82 ± 0.28a 20.45 ± 0.40c 17.20 ± 0.35e	0.317 ± 0.005bc 0.292 ± 0.007d 0.245 ± 0.004e 0.348 ± 0.004a 0.317 ± 0.007bc 0.275 ± 0.005d	9.54 ± 0.21b 6.84 ± 0.49d 4.16 ± 0.23f 11.77 ± 0.34a 8.71 ± 0.358bc 6.08 ± 0.03de	1.42 ± 0.04c 0.99 ± 0.05gh 0.56 ± 0.03j 1.75 ± 0.04b 1.25 ± 0.04de 0.83 ± 0.06i	9.50 ± 1.00 cd 6.42 ± 0.75f 4.55 ± 0.95 g 11.50 ± 1.00b 8.57 ± 1.44de 6.35 ± 0.96f	0.46 ± 0.009abc 0.37 ± 0.02ef 0.28 ± 0.01 g 0.49 ± 0.017ab 0.43 ± 0.018cde 0.34 ± 0.007f	0.068 ± 0.001def 0.054 ± 0.002gh 0.039 ± 0.002i 0.073 ± 0.002bcd 0.061 ± 0.002 fg 0.048 ± 0.004 h
**SG 65**	C S_1_ S_2_ MLE MLE + S_1_ MLE + S_2_	18.70 ± 0.33d 15.20 ± 0.57f 13.47 ± 0.21 g 22.07 ± 0.29b 18.00 ± 0.46de 15.07 ± 0.22f	0.322 ± 0.010b 0.289 ± 0.014d 0.285 ± 0.006d 0.365 ± 0.009a 0.321 ± 0.009b 0.297 ± 0.006 cd	9.35 ± 0.43b 6.62 ± 0.29d 5.30 ± 0.18e 11.44 ± 0.40a 8.38 ± 0.21c 6.55 ± 0.26d	1.45 ± 0.06c 1.18 ± 0.06ef 0.87 ± 0.04hi 1.97 ± 0.04a 1.37 ± 0.05 cd 1.05 ± 0.05 fg	10.65 ± 0.47bc 8.05 ± 0.64de 4.30 ± 0.29 g 13.62 ± 0.47a 11.20 ± 0.50b 7.22 ± 0.31ef	0.50 ± 0.022a 0.44 ± 0.03bcd 0.39 ± 0.018def 0.52 ± 0.024a 0.46 ± 0.017abc 0.43 ± 0.018bcd	0.080 ± 0.002b 0.078 ± 0.003bc 0.065 ± 0.004ef 0.089 ± 0.003a 0.076 ± 0.002bcd 0.070 ± 0.003cde
**Varieties (V)**		***	ns	*	ns	ns	ns	**
**Moringa Leaf Extract (MLE)**		ns	ns	ns	ns	ns	ns	ns
**Salinity (S)**		ns	ns	ns	ns	ns	ns	ns
**V × MLE**		**	ns	**	*	**	**	*
**V × S**		***	ns	*	ns	*	ns	ns
**MLE × S**		***	**	**	**	**	*	*
**V× MLE × S**		***	**	***	***	***	***	***

Values are means ± SE of three replicates. Different letters indicate significant responses whereas those followed by the same letters depict non-significant responses for the respective parameters at Post Hoc Duncan’s multiple range test (*p* ≤ 0.05). C; Control, S_1_; salinity level 1, S_2_; salinity level 2, MLE; moringa leaf extract, FWT; fresh weight, DWT; dry weight. *, **, and *** indicate significant differences at *p* ≤ 0.05, 0.01, and 0.001 and ns indicates non-significant differences

### Effect of MLE on K^+^ and Na^+^ concentrations of salinized rice cultivars

**Fig. 1:**
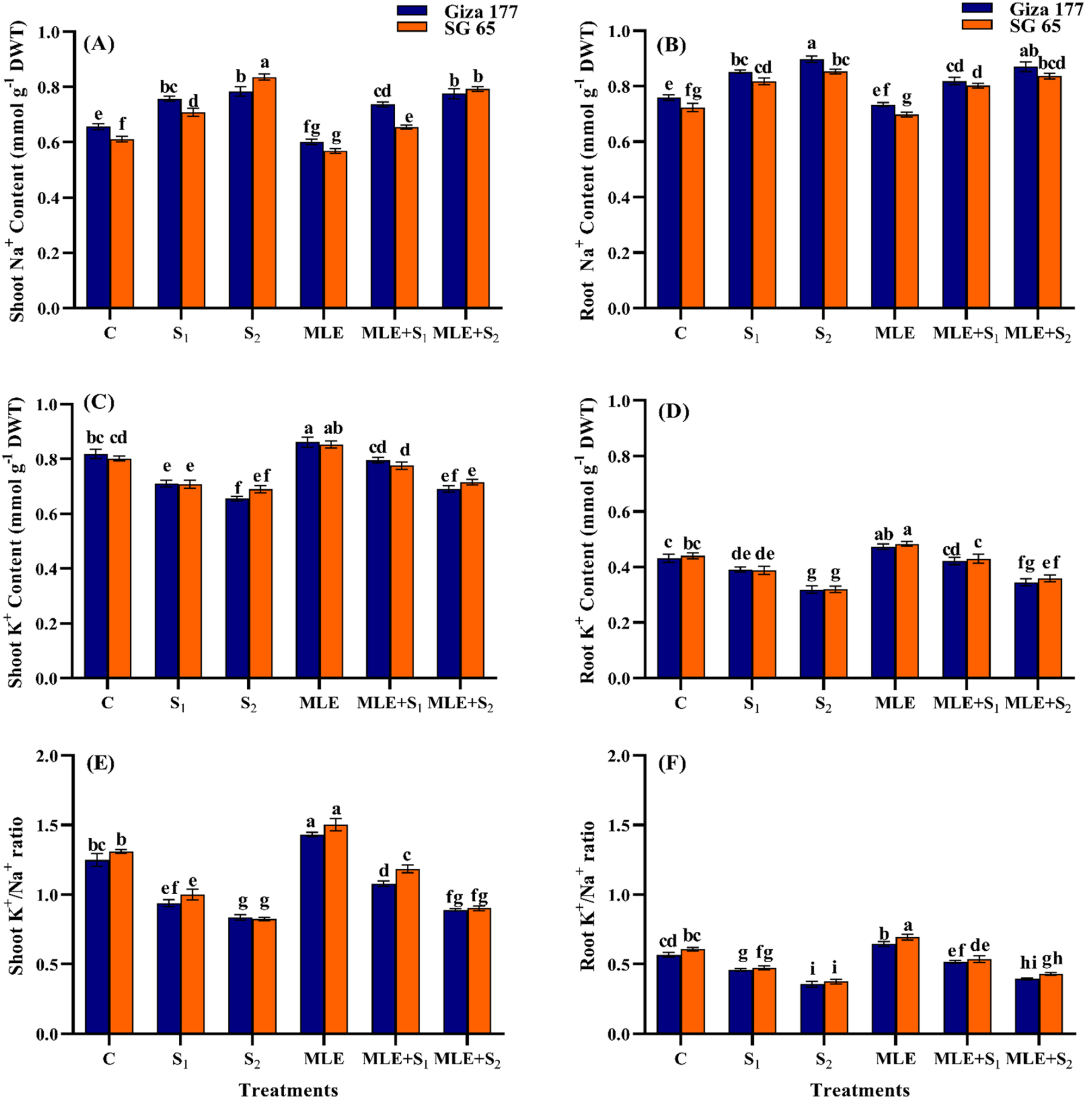
Effect of seawater stress (S) and moringa leaf extract (MLE) on Na^+^ and K^+^ content of shoots and roots (**A**) shoot Na^+^ content, (**B**) root Na^+^ content, (**C**) shoot K^+^ content, (**D)** root K^+^ content, **(E)** shoot K^+^/Na^+^ ratio, and **(F)** root K^+^/Na^+^ ratio, at vegetative stage. Values are the means ± SE of three replicates. Different letters indicate significant responses whereas those followed by the same letters indicate non-significant responses for the respective parameters at Post Hoc Duncan’s multiple range test (*p* ≤ 0.05). C; Control, S_1_; salinity level 1, S_2_; salinity level 2, MLE; moringa leaf extract

In both cultivars, salinity induced significant accumulation of Na^+^ in shoot and root (*p* ≤ 0.05) (Fig. 1). S₁ caused ~ 15.70% increase in shoot Na⁺ for both Giza 177 and SG 65, while S₂ resulted in increases of 19.39% and 36.62%, respectively, compared to controls. In roots, Na⁺ content increased by ~ 12.66% under S₁ and ~ 18.04% under S₂ in both cultivars. Conversely, salinity stress reduced K⁺ concentration and the K⁺/Na⁺ ratio in roots and shoots, particularly at S₂ (Fig. 1). Compared to controls, S₂ caused a 19.99% reduction in shoot K⁺ in Giza 177 and 13.98% in SG 65, leading to a 33% reduction in shoot K⁺/Na⁺ ratio in Giza 177 and 37% in SG 65. In roots, S₂ led to a ~ 26.56% drop in K⁺ concentration, translating to a ~ 37.72% drop in K⁺/Na⁺ ratio for both cultivars. Under non-saline conditions, MLE decreased Na⁺ levels by ~ 7.70% in shoots and ~ 3.42% in roots for both varieties compared to unprimed controls. MLE also increased K⁺ content by ~ 5.86% and ~ 14.66% and improved the K⁺/Na⁺ ratio by ~ 10.03% and ~ 13.97% in the shoot and root of Giza 177 and SG 65, respectively. Under salinity stress, MLE dramatically reduced Na⁺ concentration in shoots and roots compared to the unprimed controls (*p* ≤ 0.05). MLE also improved the K⁺/Na⁺ ratio in shoots of salinity-stressed plants by 14.90% in Giza 177 and 18.46% in SG 65 under S₁, and by 6.52% in Giza 177 and 9.40% in SG 65 under S₂. In roots, MLE induced a ~ 12.76% rise in K⁺/Na⁺ ratio under S₁ in both cultivars and increases of 11.26% in Giza 177 and 14.26% in SG 65 under S₂ (Fig. 1).

### Effect of MLE on photosynthetic pigments of salinized rice cultivars

**Fig. 2:**
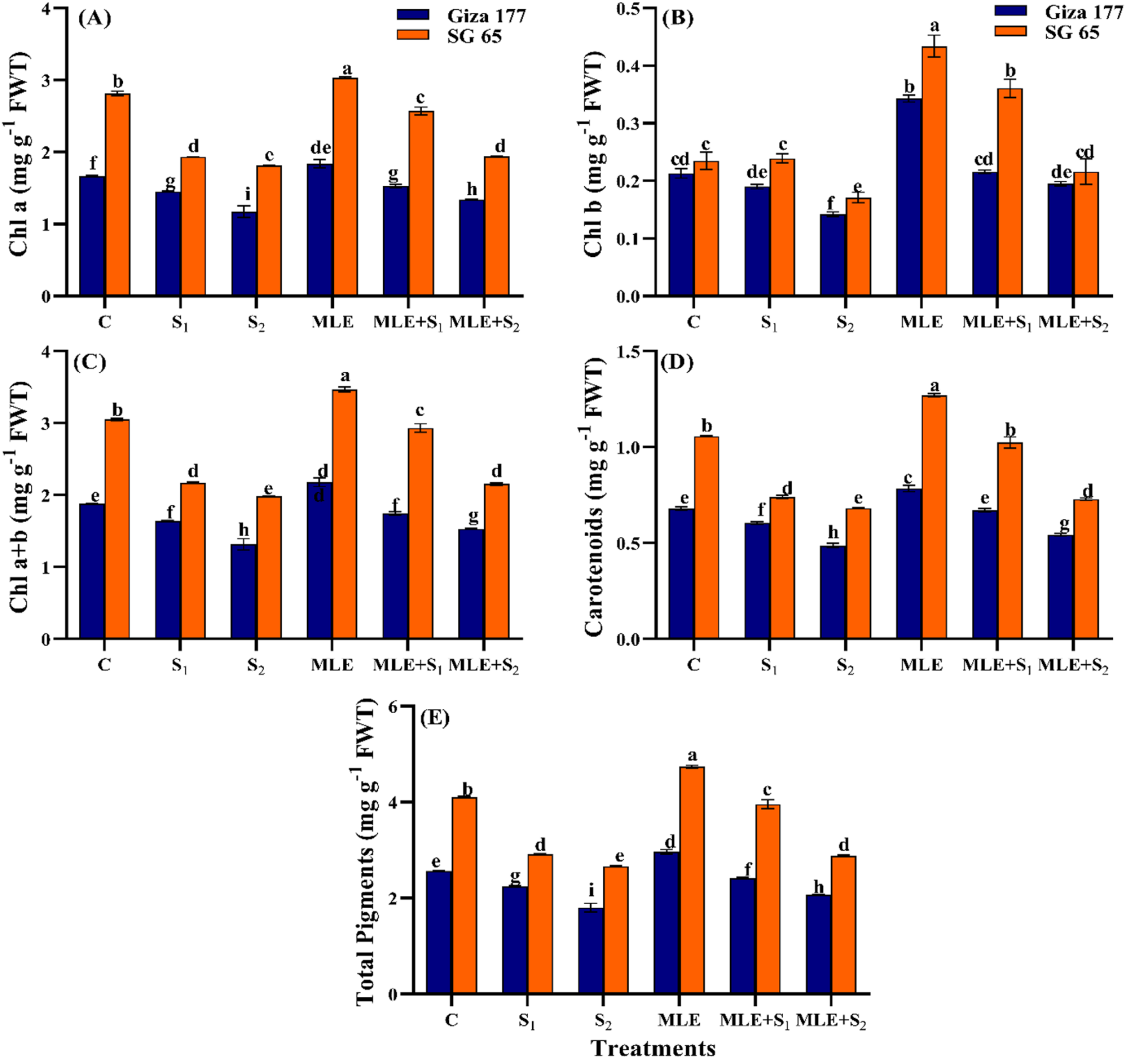
Effect of seawater stress (S) and moringa leaf extract (MLE) on photosynthetic pigments (**A**) Chl a, (**B**) Chl b, (**C**) Chl a + b, (**D**) Carotenoids, and (**E**) Total pigments in Giza 177 and SG 65 rice cultivars at vegetative stage. Values are the means ± SE of three replicates. Different letters indicate significant responses whereas those followed by the same letters indicate non-significant responses for the respective parameters at Post Hoc Duncan’s multiple range test (*p* ≤ 0.05). C; Control, S_1_; salinity level 1, S_2_; salinity level 2, MLE; moringa leaf extract

Photosynthetic pigments in both cultivars decreased significantly with increasing salinity (Fig. 2). SG 65 had higher levels of Chl a, Chl b, Chl a + b, carotenoids, and total pigments than Giza 177 across all treatments. Under S₂, reductions approached ~ 30% for Giza 177 and ~ 35% for SG 65, with Chl b being the least affected, especially in SG 65. Under non-stressed conditions, MLE significantly increased (*p* ≤ 0.05) all photosynthetic pigments in both cultivars compared to unprimed controls, with increases about 1.5 folds higher in SG 65 than in Giza 177. In the salinity-stressed plants, MLE priming minimized stress-induced reductions in photosynthetic pigments compared to the stressed unprimed plants. Under S₁ and S₂, Giza 177 showed increases of 5.42% and 14.12% in Chl a, 13.99% and 37.91% in Chl b, 10.96% and 11.44% in carotenoids, and 7.63% and 15.06% in total chlorophyll, respectively. SG 65 showed increases of 32.99% and 7.04% in Chl a, 51.11% and 26.27% in Chl b, 38.21% and 6.84% in carotenoids, and 35.80% and 8.23% in total chlorophyll, respectively. Chl b showed the maximum increase under both S₁ and S₂ in Giza 177 (14% and 38%) and SG 65 (51% and 26%).

#### Effect of MLE on gas exchange parameters of salinized rice cultivars

**Fig. 3:**
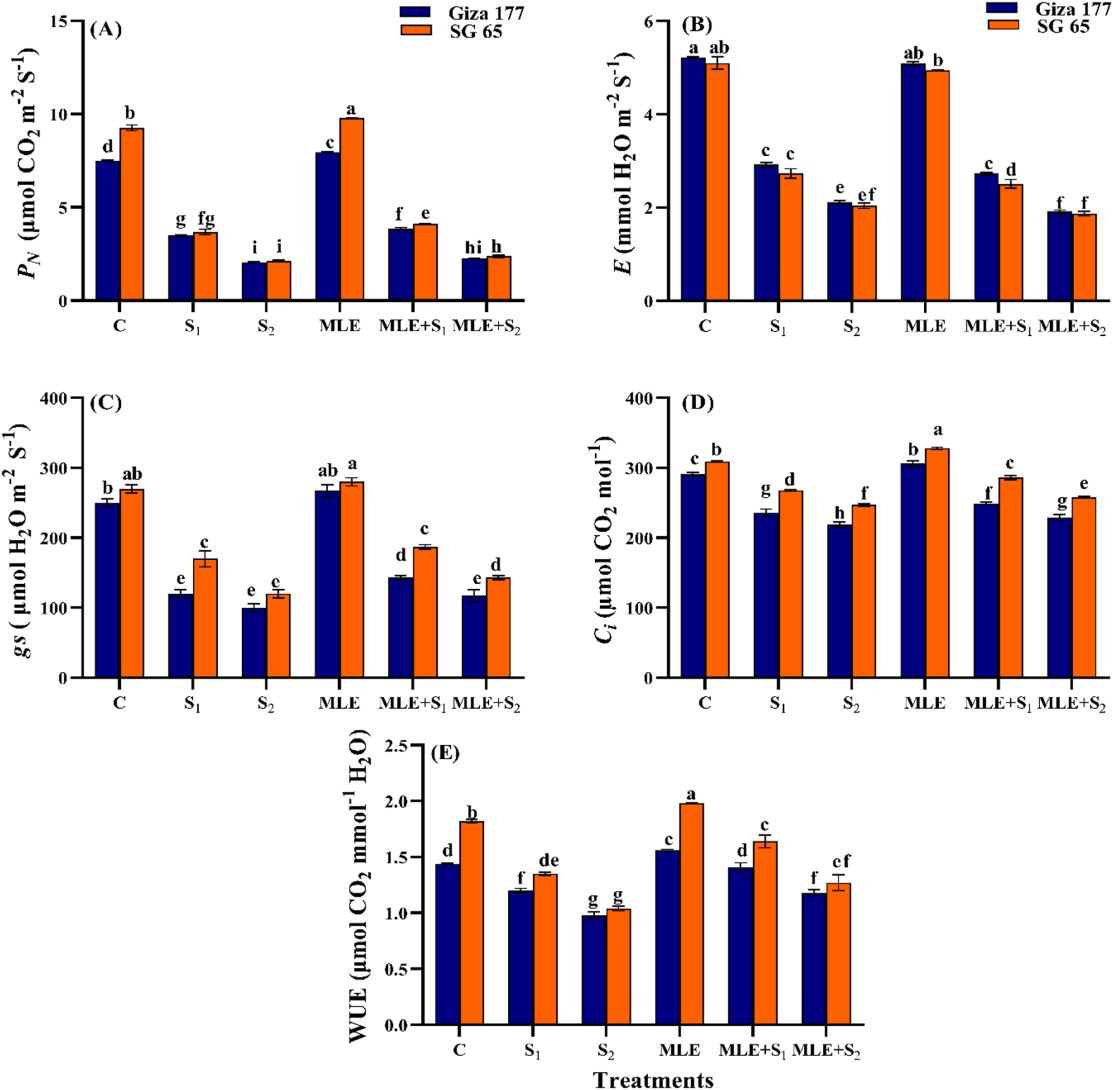
Effect of seawater stress (S) and moringa leaf extract (MLE) on gas exchange characteristics (**A**) photosynthetic rate (*P*_*N*_), (**B**) transpiration rate (*E*), (**C**) stomatal conductance (*g*_*s*_), (**D)** internal CO_2_ (*C*_*i*_), and (**E**) water use efficiency (WUE) in Giza 177 and SG 65 rice cultivars at vegetative stage. Values are the means ± SE of three replicates. Different letters indicate significant responses whereas those followed by the same letters indicate non-significant responses for the respective parameters at Post Hoc Duncan’s multiple range test (*p* ≤ 0.05). C; Control, S_1_; salinity level 1, S_2_; salinity level 2, MLE; moringa leaf extract

Salinity stress, particularly at S₂, significantly decreased (*p* ≤ 0.05) *P*_*N*_, *E,g*_*s*_, *C*_*i*_, and WUE compared to control plants (Fig. 3). S_2_ caused relatively similar reductions in *P*_*N*_, and *E* in both cultivars, however, its-induced reductions in *g*_*s*_, and *C*_*i*_ were 60%, and 24%, respectively in Giza 177 compared to 55%, and 19% in SG 65. Both cultivars had similar WUE under S_2_. MLE priming increased *P*_*N*_ and *C*_*i*_ by ~ 5.8% and WUE by ~ 9% in both cultivars, though its effect on *E* and *g*_*s*_ was non-significant compared to controls. S₁ also reduced these characteristics, but less than S₂. MLE alleviated the inhibitory effects of salinity on gas exchange components, with varying magnitudes in both cultivars. Under S₁ and S₂, MLE increased *P*_*N*_ by ~ 9.6%, *g*_*s*_ by ~ 18%, *C*_*i*_ by ~ 5.1%, and WUE by ~ 19% in Giza 177 while in SG 65, the corresponding increases were *P*_*N*_ by ~ 11.8%, *g*_*s*_ by 10% and 19.4%, *C*_*i*_ by 6.7% and 4.4%, and WUE by ~ 22%. MLE priming had a non-significant effect on *E* under salinity conditions in both cultivars (Fig. 3).

### Effect of MLE priming on carbohydrate fractions of salinized rice cultivars

**Fig. 4:**
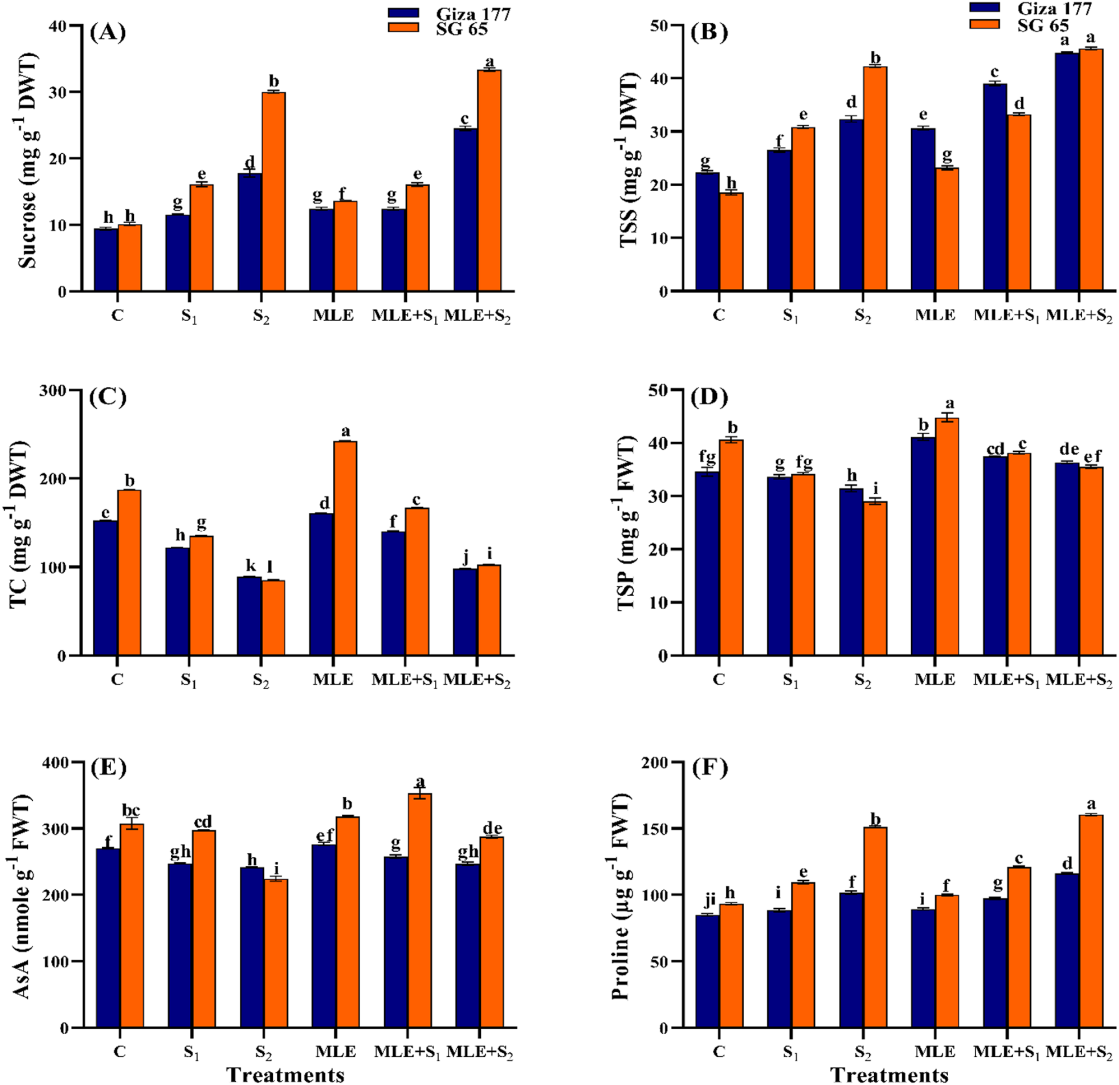
Effect of seawater stress (S) and moringa leaf extract (MLE) on carbohydrates fractions (**A**) sucrose, (**B**) total soluble sugars (TSS), (**C**) total carbohydrates (TC), as well as (**D)** total soluble protein (TSP), **(E)** ascorbic acid, and **(F)** proline in Giza 177 and SG 65 rice cultivars at vegetative stage. Values are the means ± SE of three replicates. Different letters indicate significant responses whereas those followed by the same letters indicate non-significant responses for the respective parameters at Post Hoc Duncan’s multiple range test (*p* ≤ 0.05). C; Control, S_1_; salinity level 1, S_2_; salinity level 2, MLE; moringa leaf extract

Salinity stress significantly increased the accumulation of sucrose and TSS in both cultivars compared to controls (*p* ≤ 0.05) (Fig. 4a and b). Under S₁, SG 65 had 2.6- and 3.5-fold higher sucrose and TSS than Giza 177, respectively. Under S₂, SG 65 maintained its superiority in these sugars with 2.2 and 2.86 folds higher than Giza 177, respectively. In contrast, salinity stress decreased TC in a dose-dependent manner, with the greatest decrease at S₂, reaching 41% in Giza 177 and 54.5% in SG 65 (Fig. 4c). Under non-saline conditions, MLE significantly increased sucrose and TSS by 31.6% and 37.1% in Giza 177, and by 34.2% and 25% in SG 65. It also increased TC by 5.5% in Giza 177 and 29.5% in SG 65. Under S₁ and S₂, MLE increased TSS by 47.1% and 38.3% in Giza 177 (*p* ≤ 0.05) and by ~ 7.7% in SG 65 compared to stressed unprimed plants (Fig. 4b). Sucrose was less affected, particularly in SG 65 under S₁. MLE-treated stressed plants showed a modest increase in TC, especially in SG 65 under S₁ (23.5%) compared to 15% in Giza 177. Overall, SG 65 had significantly higher levels of sucrose and TC than Giza 177 under salinity and MLE treatments.

#### Effect of MLE priming on osmolytes accumulation in salinized rice cultivars

Salinity induced a significant decline in TSP and AsA content, with greater reductions under S₂ compared to S₁ in both cultivars (Fig. 4d-f). The S₂-induced increases in these two parameters in SG 65 were 2.78 folds higher than in Giza 177.Interestingly, SG 65 had 4.21- and 3.18-fold higher proline than Giza 177 under S₁ and S₂. In non-stressed plants, MLE raised TSP and proline by 19% and 5.1% in Giza 177, and by 10.3% and 7% in SG 65, with no significant changes in AsA content. In salinity-stressed plants, MLE priming had differential effects on TSP, proline, and AsA content. Under S₁, MLE increased TSP and proline by ~ 10.75% in both cultivars, while AsA was minimally affected in Giza 177 (3%) and increased by 18.8% in SG 65. Under S₂, MLE induced ~ 14.5% increases in TSP and proline in Giza 177, and 22.3% and 6% in SG 65, respectively. Overall, MLE had a greater stimulative effect on TSP and AsA content in SG 65 leaves than in Giza 177, particularly under S₂. However, Giza 177 leaves contained significantly higher proline levels than SG 65 under S₂.

#### Effect of MLE on oxidative stress indicators of salinized rice cultivars

**Fig. 5:**
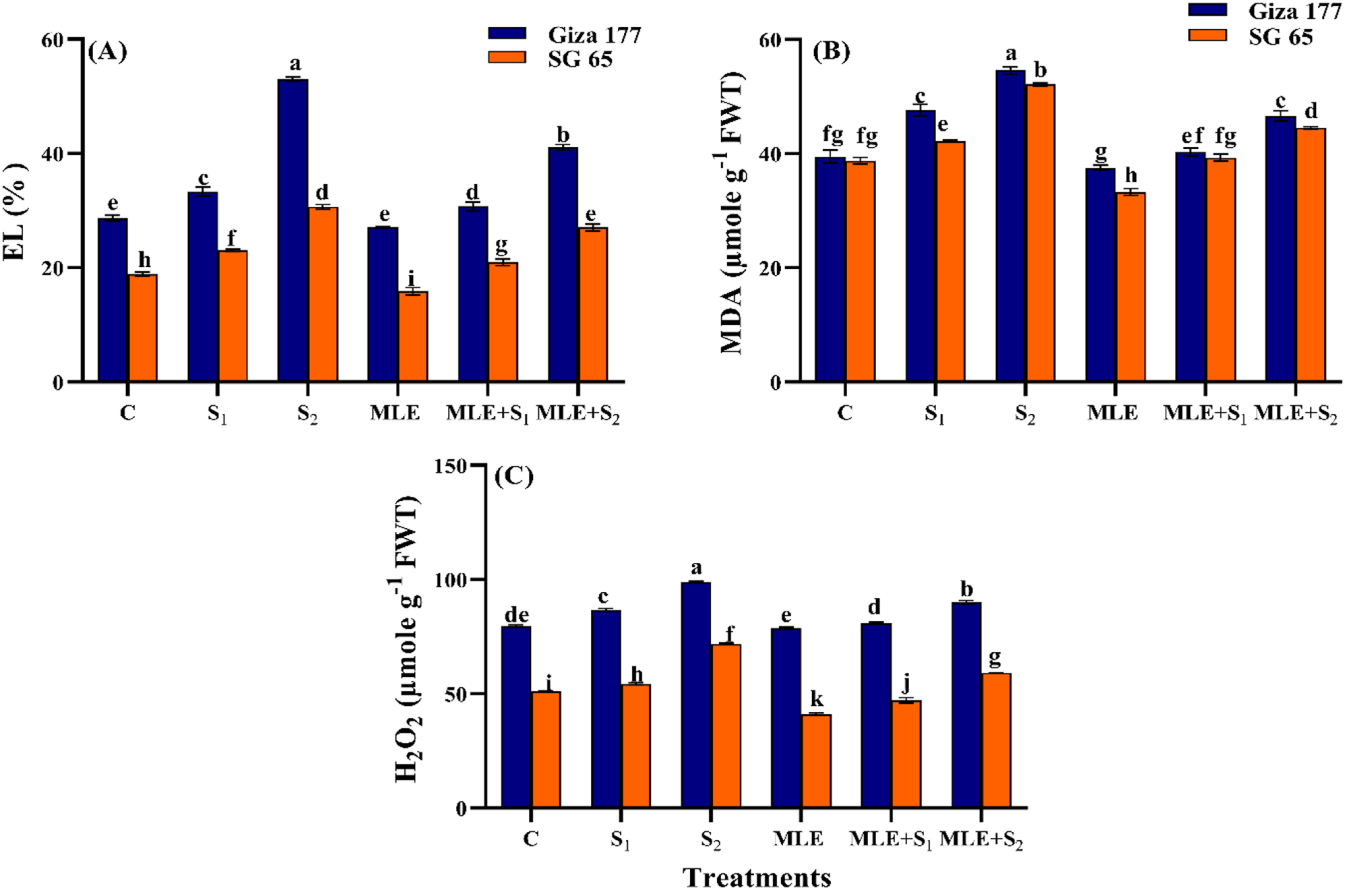
Effect of seawater stress **(S)** and moringa leaf extract (MLE) on oxidative stress markers **(A)** Electrolyte leakage (EL), **(B)** Malondialdehyde (MDA), and **(C)** hydrogen peroxide (H_2_O_2_) in Giza 177 and SG 65 rice cultivars at vegetative stage. Values are the means ± SE of three replicates. Different letters indicate significant responses whereas those followed by the same letters indicate non-significant responses for the respective parameters at Post Hoc Duncan’s multiple range test (*p* ≤ 0.05). C; Control, S_1_; salinity level 1, S_2_; salinity level 2, MLE; moringa leaf extract

Salinity stress increased MDA, H_2_O_2_, and EL in both rice cultivars in a dose-dependent manner, relative to controls (Fig. 5). S_2_ elicited increases of 24.21% and 40.3% in H_2_O_2_, 38.16 and 34.5% in MDA, and 85.2 and 61.89% in EL in Giza 177 and SG 65, respectively. Yet, SG 65 maintained a higher average than Giza 177, particularly in H_2_O_2_. The S_1_-induced increments in these oxidative stress markers were significantly smaller in both cultivars. Under normal conditions, MLE priming either modestly lowered or did not modify these biomarkers with SG 65 having significantly lower concentrations than Giza 177. Under low salinity level, MLE priming reduced MDA by 15.3 and 6.8%, H_2_O_2_ by 6.7% and 13.3%, and EL by ~ 8.35%, in Giza 177 and SG 65, respectively. Under high salinity level, the corresponding MLE-induced reductions were ~ 14.6% in MDA, 9% and 17.7% in H_2_O_2_, 22% and 11.8% in EL, in Giza 177 and SG 65, respectively, compared to their unprimed peers (Fig. 5).

#### Effect of MLE on the activity of antioxidant enzymes of salinized rice cultivars

**Fig. 6:**
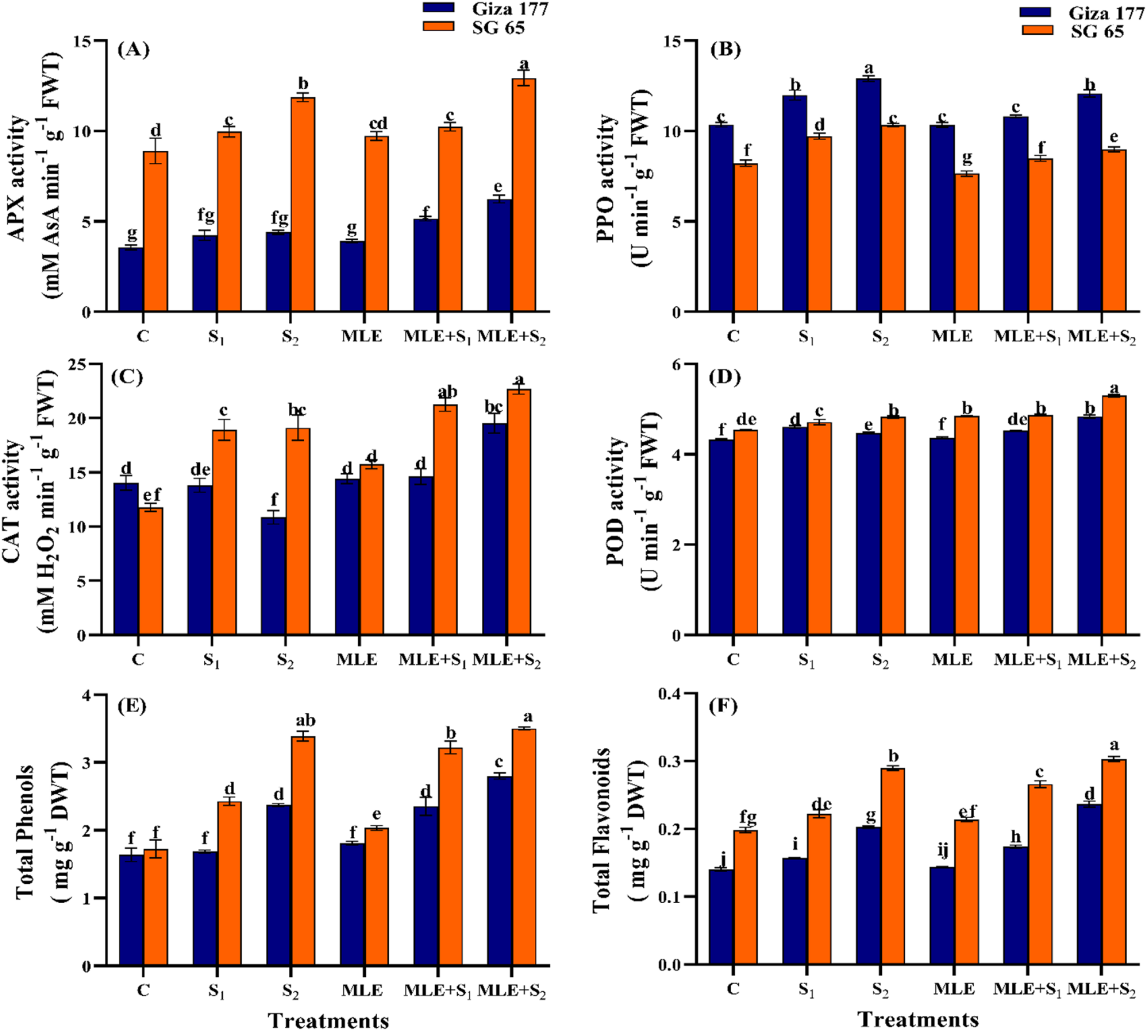
Effect of seawater stress (S) and moringa leaf extract (MLE) on the antioxidant system (**A**) ascorbate peroxidase (APX), (**B**) polyphenol oxidase (PPO), (**C**) catalase (CAT), (**D)** peroxidase (POD), **(E)** total phenols, and **(F)** total flavonoids in Giza 177 and SG 65 rice cultivars at vegetative stage. Values are the means ± SE of three replicates. Different letters indicate significant responses whereas those followed by the same letters indicate non-significant responses for the respective parameters at Post Hoc Duncan’s multiple range test (*p* ≤ 0.05). C; Control, S_1_; salinity level 1, S_2_; salinity level 2, MLE; moringa leaf extract

Giza 177 and SG 65 showed differential activities of the tested antioxidant enzymes in response to salinity. In SG 65, APX activity increased significantly by 33.3% as salinity increased while no statistical difference was detected in APX activity in Giza 177 (*p* ≤ 0.05) (Fig. 6a-d). SG 65 had 2-4-fold higher APX activity than Giza 177 across normal and stress conditions. Unlike APX, PPO activity increased in both cultivars as salinity increased with Giza 177 having significantly higher PPO activity than SG 65 under normal and stress conditions. Salinity suppressed CAT activity in Giza 177, particularly at the S_2_ level (23%), but CAT activity increased to relatively similar levels under S_1_ and S_2_ in SG 65 (~ 61.6%). POD exhibited the least difference among Giza 177 and SG 65 cultivars with a slight increase in both in response to salinity. Compared to their unprimed peers, MLE priming induced a significant increase in APX activity of Giza 177 under S_2_ only (42%). MLE decreased the salinity-induced increase in PPO activity in both cultivars, relative to the unprimed control. Furthermore, MLE increased CAT activity by 80% in Giza 177 only under S_2_, whereas in SG 65, MLE induced a significant increase in CAT activity under both salinity levels (~ 15.5%). For POD, MLE induced a slight increase in its activity in Giza 177 only under the S_2_ level, whereas in SG 65, MLE increased POD activity across normal and salinity treatments. Overall, the SG 65 cultivar consistently showed higher antioxidant activity than Giza 177 across all treatments.

### Effect of MLE on antioxidant compounds of salinized rice cultivars

Both cultivars had relatively similar phenolic concentrations under non-stress conditions. Salinity increased remarkably total phenolic content in Giza 177 by 45% under S_2_ only, while in SG 65, total phenolic content increased by 41% at S_1_ and 97% at S_2_, respectively (Fig. 6e). Compared to their unprimed peers, MLE-treated SG 65 plants had significantly higher phenolics across all treatments, but these responses were noted only in Giza 177-stressed plants. Unlike phenolics, Giza 177 had significantly lower flavonoids than SG 65 under non-stress conditions (Fig. 6f). S_2_-stressed plants of both cultivars accumulated higher levels of flavonoids (~ 45.1%). MLE induced flavonoid accumulation under normal and stressed conditions compared to their unprimed plants.

### Principal component analysis (PCA) and correlation studies

**Fig. 7:**
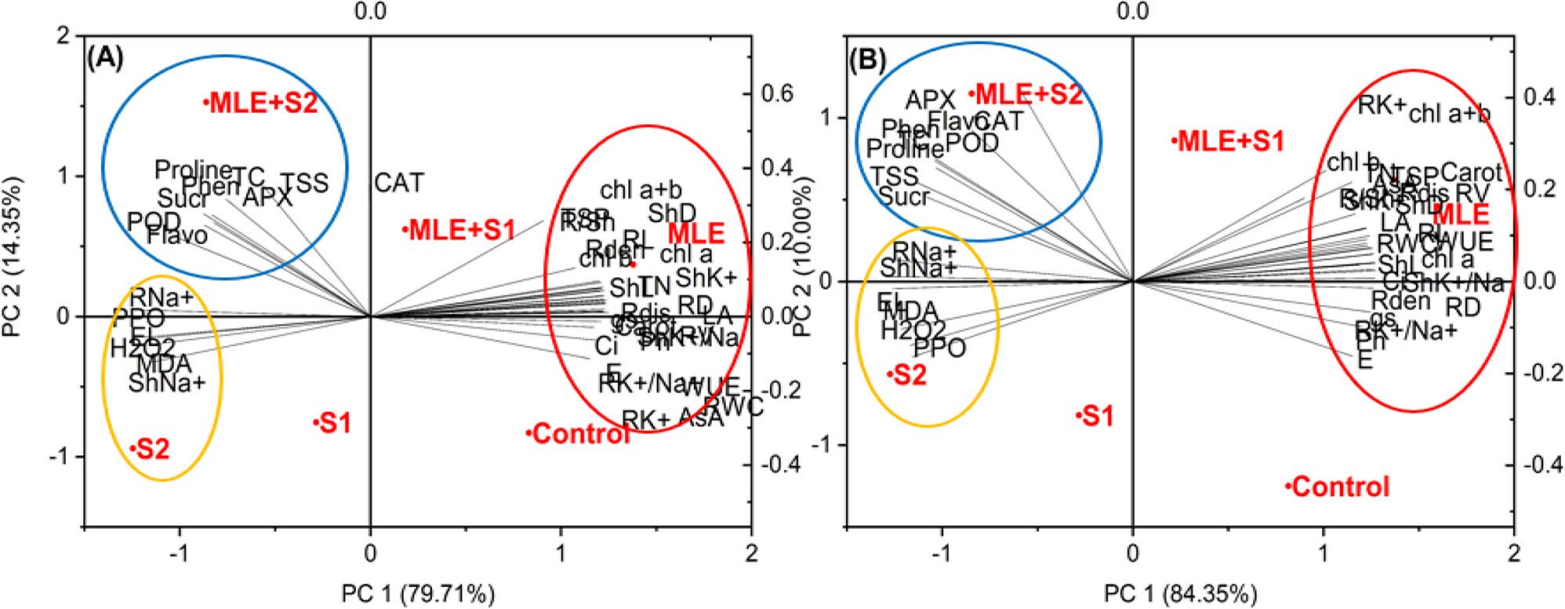
Principal component analysis (PCA) of growth, physiological, and biochemical traits in (**A**) Giza 177 and (**B**) SG 65 plants. *Abbreviations:* ShL; Shoot length, ShD; Shoot dry weight, TN;Tillers number, LA; Leaf Area, RWC; Relative Water Content, RL; Root Length, RL/SL ratio; Root/Shoot ratio, RD; Root dry weight, RV; Root Volume, Rdis; Root distribution, Rdes; Root density, *Pn*; photosynthetic rate, *E*; transpiration rate, *Ci*; internal CO_2_, *g*_*s*_; stomatal conductance, WUE; Water Use Efficiency, Chl a; Chlorophyll a, Chl b; Chlorophyll b, Carot; Carotenoids, TC; Total Carbohydrates, Suc; Sucrose, TSS; Total Soluble Sugars, AsA; Ascorbic Acid, TSP; Total Soluble Protein, H_2_O_2_; Hydrogen Peroxide, MDA; Malondialdehyde, EL; Electrolyte Leakage, APX; Ascorbate Peroxidase, CAT; Catalase, POD; Peroxidase, PPO; Polyphenol Oxidase, Phen; Phenols, Fl; Flavonoids, Sh Na^**+**^; Shoot Na^+^, Sh K^**+**^; Shoot K^+^,Sh K^**+**^/Na^**+**^ratio; Shoot K^+^/Na^+^ ratio, R Na^**+**^; Root Na^+^, R K^**+**^; Root K^+^, R K^**+**^/Na^**+**^ratio; Root K^+^/Na^+^ ratio

**Fig. 8:**
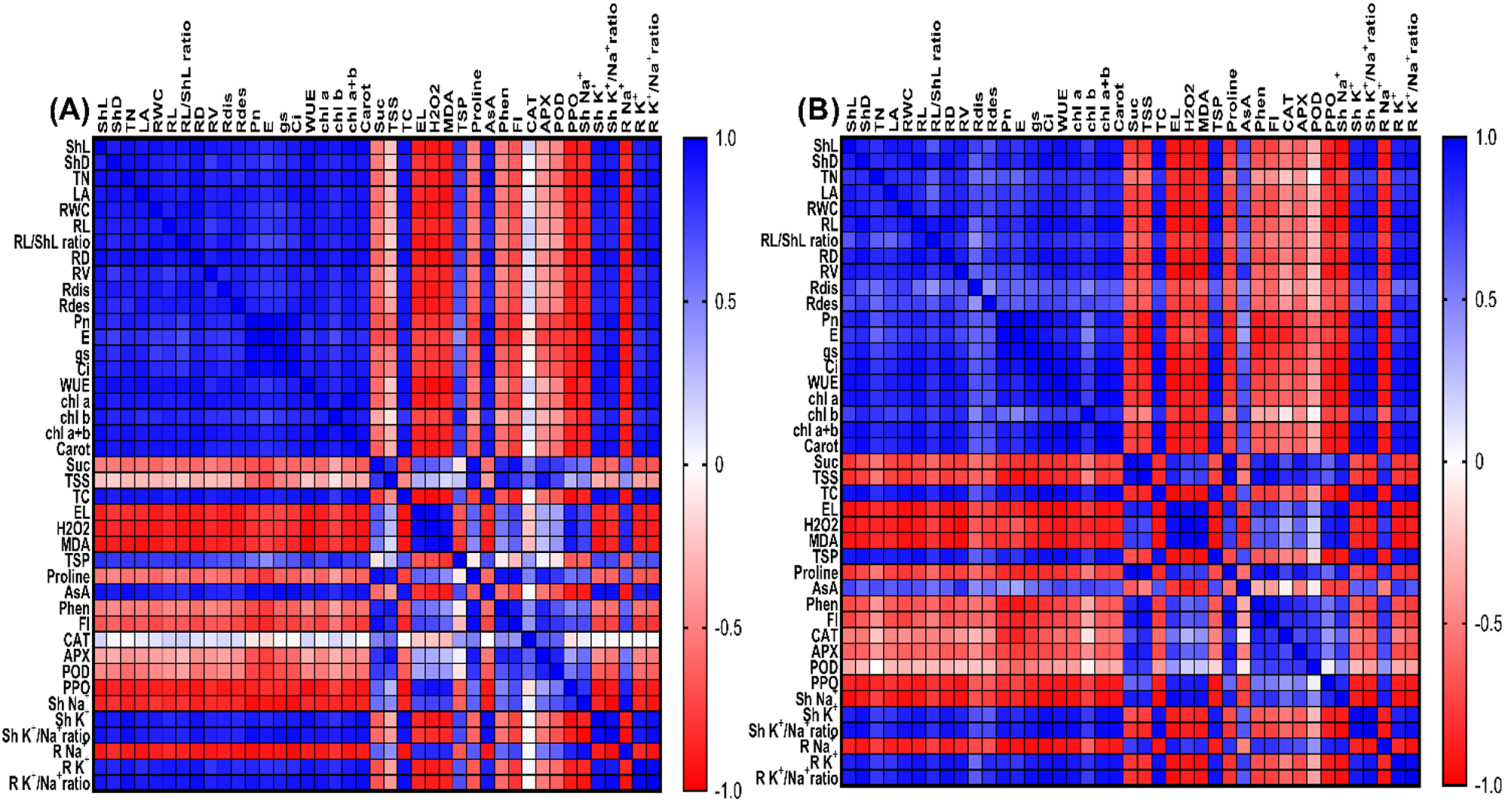
Heatmap of Pearson’s correlation analysis of some investigated traits in MLE treated and non-treated **(A)** Giza 177 and **(B)** SG 65 plants grown under seawater stress. Blue and red colors represent positive and negative correlations, respectively, according to the color scale. *Abbreviations:* ShL; Shoot length, ShD; Shoot dry weight, TN;Tillers number, LA; Leaf Area, RWC; Relative Water Content, RL; Root Length, RL/ShL ratio; Root/Shoot ratio, RD; Root dry weight, RV; Root Volume, Rdis; Root distribution, Rdes; Root density, *Pn*; photosynthetic rate, *E*; transpiration rate, *Ci*; internal CO_2_, *gs*; stomatal conductance, WUE; Water Use Efficiency Chl a; Chlorophyll a, Chl b; Chlorophyll b, Carot; Carotenoids, TC; Total Carbohydrates, Suc; Sucrose, TSS; Total Soluble Sugars, AsA; Ascorbic Acid, TSP; Total Soluble Protein, H_2_O_2_; Hydrogen Peroxide, MDA; Malondialdehyde, EL; Electrolyte Leakage, APX; Ascorbate Peroxidase, CAT; Catalase, POD; Peroxidase, PPO; Polyphenol Oxidase, Phen; Phenols, Fl; Flavonoids, Sh Na^+^; Shoot Na^+^, Sh K^+^; Shoot K^+^,Sh K^+^/Na^+^ratio; Shoot K^+^/Na^+^ ratio, R Na^+^; Root Na^+^, R K^+^; Root K^+^, R K^+^/Na^+^ratio; Root K^+^/Na^+^ ratio

PCA was executed on the tested parameters to explore relationships among different variables across treatments (Fig. 7). Two principal components (PC1 and PC2) were extracted with eigenvalues of more than 1, accounting for ~ 94.19% of the variability in the two cultivars. PC1 explained 78.80% and 84.20%, while PC2 revealed 15.02% and 10.35% of the total variation in Giza 177 and SG 65, respectively. In both cultivars, three clusters were obtained in the biplot: the first cluster included oxidative stress markers (MDA, H_2_O_2_, EL), shoots and roots’ Na^+^ contents, and PPO enzyme. These parameters were closely associated with high salinity level (S_2_). The second cluster demonstrated the positive effects of MLE alone on growth parameters (shoot length, shoot dry weight, number of tillers, leaf area, RWC, root length, root fresh weight, root dry weight, root/shoot ratio, root volume, root distribution, and root density), photosynthetic pigments (Chl a, Chl b, Chl a + b, and carotenoids), gas exchange parameters (*P*_*N*_, *E,g*_*s*_, *C*_*i*_, WUE), and AsA in two rice cultivars. The third cluster included proline, CAT (in SG 65), POD, APX, total phenols, flavonoids, TSP, TTS, sucrose, TC in both rice cultivars under high salinity level and MLE effect. These metrics demonstrated a strong correlation with the MLE + S_2_ treatment, particularly in the SG 65 cultivar, suggesting an effective role of MLE in ameliorating the salinity-induced damages. Pearson’s correlation analysis (Fig. 8) revealed positive correlations among the tested growth traits, root attributes, photosynthetic pigments, gas exchange parameters, TSP, AsA, TC, shoot and root K^+^ content, and K^+^/Na^+^ in both cultivars. Conversely, these morphological and physiological parameters exhibited strong negative correlations with stress markers (MDA, H₂O₂, EL, proline), CAT (in SG 65 cultivar), POD, APX, PPO, sucrose, TSS, total phenols, flavonoids, and shoot and root Na^+^ content.

## Discussion

Developing novel rice genotypes with enhanced salt tolerance alongside the application of biostimulants with growth-promoting and stress-alleviating potential is an effective approach for improving rice growth and productivity in salt-affected lands [56, 57]. Herein, we examined the growth and physiological responses of Giza 177 and a new introgression line (*sativa*/*glaberrima*; SG 65) under low and high salinity stress with and without MLE priming. Our growth analysis revealed differential reductions in plant growth in both cultivars under salt stress, with SG 65 outperforming Giza 177 in most growth indices, including plant biomass, leaf area, number of tillers, root/shoot ratio, and other root traits (volume, distribution, and density) (Tables 2 and 3). Such responses were associated with comparable differential alterations in RWC in both cultivars. These growth retardations coincide with the reported salinity-induced growth inhibitions in various rice varieties [58]. Such salinity-elicited growth retardations are attributed to ions accumulation (particularly Na^+^) within plant tissues, disrupting water balance, stunting cell elongation and division, and ultimately hindering overall plant growth. Additionally, reduced leaf area and fewer tillers may be adaptive mechanisms in both cultivars via avoiding excessive water loss under salinity and retaining toxic ions in the roots rather than the aerial parts [59, 60]. The superior SG 65 growth indicates greater plasticity in its morphological traits and better modulation of osmotic pressure [61]. These SG 65’s responses are likely influenced by *O. glaberrima*-derived genes, as efficient osmotic adjustment, particularly in the shoot, has been documented in several *O. glaberrima* accessions [62]. The improved SG 65’s growth mimics that of salt-tolerant genotypes and implies efficient adaptive physiological mechanisms under salinity stress [61]. Priming Giza 177 and SG 65 seeds in MLE significantly enhanced growth in both cultivars and ameliorated the salinity-induced damages under stressful conditions with SG 65 being more responsive. These findings agree with the reported MLE-induced growth improvements in various plants including organic fennel [63], and geranium [64]. The MLE promotive effects are attributed to its multifaceted positive impacts on salt tolerance-related physiological and biochemical signaling pathways. Its IAA, cytokinins, and zeatin content drive these effects and promote cell division and elongation, increasing growth and biomass accumulation during salinity stress. Further, the presence of Ca^2+^, K^+^, Mn^2+^, Fe^2+^, Mg^2+^, and Zn^2+^ in MLE could have contributed to boosting growth in both cultivars [65].

In both cultivars, salt stress increased Na^+^ while decreased K^+^ levels and the K^+^/Na^+^ ratio in their shoots and roots with SG 65 having better records than Giza 177 (Fig. 1). Similar responses have been reported in rice [66, 67], lettuce, spinach, and common purslane [68]. Such salinity-induced ionic responses disrupt ion homeostasis in Giza 177 and SG 65 tissues, causing physiological disorders, and leading to ion toxicity and growth hindrance in both varieties (Tables 2 and 3) [69–71]. The response of SG 65, like many *O. glaberrima *accessions and other salt-tolerant rice cultivars, is associated with reduced Na ^+^ transport to the shoot and limited Na ^+^ deposition in leaves, possibly through a high expression of OSHKT1;5 gene, located within the Saltol QTL on chromosome 1, thereby improving the shoot K^+^/Na^+^ ratio [72–74]. The enhanced tolerance to salt-induced Na^+^ accumulation in SG 65, as evidenced by its improved growth under salt stress, is likely driven by genes of *O. glaberrima* in which ion tolerance mechanisms are integral to maintaining overall plant performance under salinity stress [62]. Consequently, the *O. glaberrima* genome endows SG 65 with key ion tolerance strategies essential for managing salinity. Additionally, the SG 65 cultivar may use tissue Na^+^ tolerance mechanisms as observed in wild rice species whereas Giza 177 lacks these mechanisms due to selection for Na^+^ exclusion [75, 76]. MLE priming decreased Na^+^ levels in the shoot while increasing Na^+^ accumulation in the root (Fig. 1) which was associated with improved growth of Giza 177 and SG 65 cultivars (Tables 2 and 3). This aligns with findings that limiting toxic ion accumulation enhances essential mineral uptake, plant metabolism, and resistance to salt stress [77]. This MLE promotive effect is attributed to its role in restricting Na^+^ uptake and enhancing K^+^ absorption, which positively influences growth through enzyme activation, osmoregulation, and selective sodium exclusion [78, 79]. Such a MLE-induced increase in K^+^/Na^+^ ratio seems to be a key route through which MLE alleviates salinity stress [80].

Salinity stress significantly reduced photosynthetic pigments, gas exchange, and WUE with more pronounced adverse effects in Giza 177 than in SG 65 (Figs. 2 and 3). These findings align with the reported negative impact of salinity on the photosynthetic machinery in rice [81], wheat, barley [82], and tomatoes [83]. In salt-stressed plants, the reduction in photosynthetic pigments is linked to the salinity-induced ionic stress, inducing the production of ROS, disrupting chloroplast integrity, and damaging the chlorophyll biosynthetic pathway [84, 85]. In addition, salinity induces chlorophyllase activity and limits Mg^2+^ uptake consequently driving chlorophyll annihilation [67]. Furthermore, salinity restricts stomatal regulation, decreases leaf water potential, and reduces gas exchange through ABA-induced stomata closure, lowering CO_2_ level, and photosynthesis. This limits photosynthates production and reduces growth in both cultivars (Tables 2 and 3) [86, 87]. Interestingly, the stressed SG 65 cultivar exhibited higher *g*_*s*_ and *C*_*i*_ than Giza 177 (Fig. 3C, D). These findings align with previously reported elevated levels of these key photosynthetic characteristics in leaves of many *O*. *glaberrima* accessions [62]. Additionally, the higher photosynthetic pigments content observed in the stressed SG 65 cultivar (Fig. 2) likely contributed to its increased carbohydrates, superior growth, and overall better performance relative to Giza 177. MLE treatment improved photosynthetic pigments and CO_2_ assimilation in non-stressed and stressed rice cultivars, with more pronounced effects in SG 65 (Figs. 2 and 3). This aligns with studies showing MLE-enhanced photosynthetic pigments and gas exchange characteristics in maize [88], quinoa [89], and rice [90]. The MLE-enhanced chlorophyll content is attributed to its Fe^2+^ and Mg^2+^, which facilitate protoporphyrin conversion to chlorophyllide, cytokinin’s role in chlorophyll synthesis and degradation, and antioxidant compounds that boost pigment content [90–92].

Saline irrigation increased EL, H_2_O_2,_ and MDA in both cultivars with higher levels in Giza 177 than SG 65 (Figs. 5, 7 and 8). This aligns with the idea that excessive ROS under salinity triggers lipid peroxidation, disruption of membrane permeability, and electrolyte outflows in rice plants [93, 94]. The significantly lower oxidative stress markers in SG 65 compared to Giza 177 indicate that SG 65 may have developed more effective ROS scavenging strategies likely driven by the African rice-derived gens. This supports SG 65’s potential as a salt-tolerant line, while Giza 177 is salt-sensitive. MLE treatment effectively decreased EL, H_2_O_2_, and MDA, particularly in SG 65 under low salt stress, demonstrating MLE’s ability to quench ROS and improve SG 65’s tolerance to oxidative stress. Similar stress-alleviating effects of MLE have been reported in sunflower [95] and wheat [96]. MLE mitigation effects are most likely driven, directly or indirectly, by its antioxidant-rich composition which protects the cell membrane and fosters a healthier metabolic state that results in crop plant growth (Supplementary Table 1) [97]. Altogether, these findings highlight the MLE’s potential to increase the salinity tolerance of rice plants.

SG 65 surpassed Giza 177 in synthesizing osmoprotectants (TSS, TC, sucrose, proline, and TSP) (Fig. 4), antioxidant enzymes (CAT, POD, and APX), and non-enzymic antioxidants (flavonoids, phenolics, and AsA), while Giza 177 had higher PPO enzyme activity (Fig. 6). These findings had been reported in other rice cultivars and suggest coordinated responses to enhance cell turgidity and osmotic balance, mitigate salt-triggered oxidative damage, and protect plants from salt-induced cellular abnormalities [98–102]. Interestingly, a strong positive correlation among proline, oxidative stress markers, non-enzymatic compounds, and antioxidant enzymes (APX, CAT, and POD) was observed in our correlation analysis (Fig. 8). The high levels of oxidative stress markers and the reduced APX and CAT activities in stressed Giza 177 compared to SG 65 (Figs. 5 and 6) suggest a more intense oxidative stress in Giza 177 than SG 65. Such intensive stress may greatly jeopardize the benefit of Giza 177’s high PPO activity which may be advantageous under low levels of oxidative stress [103]. In contrast, the higher levels of CAT, POD, and APX in SG 65 reflect higher ROS scavenging activity thus reducing their deleterious effects on photosynthetic machinery, membrane stability, and cellular biomolecules [104, 105]. The elevated ROS scavenging capacity is likely attributed to African rice genes, which may enhance the activity of CAT, POD, and APX, thereby efficiently neutralizing ROS.

MLE treatment enhanced the accumulation of osmolytes in both cultivars under control and stressed conditions (Fig. 4), consistent with the reported MLE-induced accumulation of AsA in lettuce [106], proteins in wheat [79], proline in safflower [65], and carbohydrates in rice [107] under salinity stress. MLE also enhanced the activity of CAT, APX, and POD, as well as the level of phenols and flavonoids, particularly in the SG 65 cultivar consistent with similar MLE-induced responses in salt-stressed wheat [108] and safflower plants [65]. In addition, the upregulation of the expression of GR, CAT, and GST by MLE has been reported in salt-stressed Plantago [109]. MLE was generally more effective under high salt stress than under low stress as shown by the strong correlation between osmolytes, antioxidants, and MLE + S_2_ treatment (Fig. 7). The MLE’s rich content of proline, ascorbate, zeatin, carotenoids, and growth-promoting substances and elements, help rice plants scavenge ROS and mitigate salt stress effects [108, 110] and via improving osmolytes and antioxidants accumulation, plant water content, photosynthesis, metabolic regulation that ultimately improve biomass and plant performance in salt-affected soils [111–113].

Although our study did not directly investigate the molecular responses of Giza 177 and SG 65 to salinity and MLE, our physiological observations such as improved growth under stress, reduced Na^+^/K^+^ ratio, and elevated antioxidant enzyme activity can be linked to established molecular mechanisms triggered by MLE. For instance, MLE has been shown to significantly upregulate the expression of genes encoding osmotin-like proteins (OLPs), thereby enhancing cellular osmotic adaptation, plant growth, and productivity in the salinity-stressed *Ocimum basilicum* [114]. Additionally, MLE activates indole acetic acid-and ethylene-responsive genes (AtIAA and PR4), and genes related to abscisic acid-, salicylic acid-, indole acetic acid- and ethylene-hormonal signaling pathways in *Arabidopsis thaliana*. These pathways shape plant growth, development, and responses to environmental stresses including salinity. However, the relative contribution of these pathways to MLE-mediated salt tolerance remains unclear [115]. The enhanced responses observed in moringa-treated SG 65, compared to Giza 177, suggest that several effective *O. glaberrima-derived* QTLs/genes are particularly responsive to components of MLE, thereby enhancing tolerance mechanisms more effectively than in Giza 177. Future molecular investigation of the MLE-induced responses will be beneficial for a better understanding of MLE’s mode of action in the two cultivars.

## Conclusion

The current study demonstrated the beneficial effects of incorporating genes from *Oryza glaberrima* into *Oryza sativa*, along with the application of MLE priming in mitigating the salinity stress-induced damage in growth and physiological processes in rice via (1) improvement of various components of the photosynthetic machinery, (2) preserving osmoprotection and boosting proline, total soluble proteins, ascorbic acid, and carbohydrates, (3) lowering oxidative stress through increasing the activities of ROS-scavenging enzymatic and non-enzymatic antioxidants; and (4) improving K^+^ uptake and decreasing Na^+^ uptake. Also, the current investigation indicates that African rice provided SG 65 cultivar with favorable growth traits and physiological responses making it more tolerant against salinity than Giza 177; particularly when primed with MLE. Altogether, the combined utilization of African rice-derived genes and MLE application could be an effective strategy for sustainable and eco-friendly improvement of rice salt tolerance in salt-affected lands. Future genomic and transcriptomic analyses are required to dissect the molecular basis of the differential growth and physiological responses of Giza 177 and SG 65 reported in the current study.

## Electronic supplementary material

Below is the link to the electronic supplementary material.

Supplementary Material 1: The results of ANOVA showing the effects of three individual factors—variety, MLE, and salinity treatment —as well as their interactions on the parameters measured in the experiment are in supplementary materials (supplementary Table 3).

## Data Availability

All data generated or analyzed during this study are included in this published article and any further inquiries can be directed to the corresponding author.
