# Supplementary material for: Enhancing salinity tolerance in cultivated rice through introgression of African rice genes and application of moringa leaf extract
Source: BMC Plant Biol. 2025 Feb 7;25:163. doi: 10.1186/s12870-025-06102-y (PMC11804035; doi:10.1186/s12870-025-06102-y)
Supplement: Supplementary file 1 — Supplementary Material 1: The results of ANOVA showing the effects of three individual factors—variety, MLE, and salinity treatment —as well as their interactions on the parameters measured in the experiment are in supplementary materials (supplementary Table 3). [file 12870_2025_6102_MOESM1_ESM.docx]

**Supplementary table 1** Concentration of different constituents in MLE

| Phytohormonal profile concentration (mg/100g FWT) | | | | |
| --- | --- | --- | --- | --- |
| IAA | GA | Zeatin | Kinetin | ABA |
| 3.005 | 8.87 | 0.213 | 0.013 | 0.022 |
| Antioxidant compounds | | | | |
| Total Phenols  (mg gallic acid g^-1^ DWT) | Total Flavonoids  (mg quercetin equivalent g^-1^ DWT) | | Ascorbic Acid  (nmole g^-1^ FWT) | |
| 5.62 | 0.695 | | 0.501 | |
| Antioxidant enzymes | | | | |
| CAT  (mM H_2_O_2_ min^-1^ g^-1^ FWT) | POD  (U min^-1^ g^-1^ FWT) | | PPO  (U min^-1^ g^-1^ FWT) | APX  (mM AsA min^-1^ g^-1^ FWT) |
| 27.59 | 5.43 | | 13.57 | 2.72 |
| Photosynthetic pigments (mg g^-1^ FWT) | | | | |
| Chl a | Chl b | Carotenoids | | Total Pigments |
| 1.436 | 0.128 | 0.571 | | 2.135 |
| Carbohydrates (mg glucose equivalent g^-1^ DWT) and total soluble proteins (mg g^-1^ FWT) | | | | |
| Total carbohydrates | Total soluble sugars | | Total soluble proteins | |
| 214 | 90.107 | | 12.33 | |

**Supplementary table 2** Some physical and chemical characteristics of soil

| **W.H.C**  **(%)** | | **CaCO_3_**  **(%)** | **TSS (%)** | **C/N ratio** | **Total**  **nitrogen**  **(%)** | **Organic**  **carbon**  **(%)** | **EC**  **(mS/cm)** | **pH** | **Soil**  **characters** |
| --- | --- | --- | --- | --- | --- | --- | --- | --- | --- |
| 50.11 | | 4.22 | 0.52 | 2.8 | 0.26 | 1.025 | 1.51 | 8.2 | **Silt soil**  **(clay: sand 2:1)** |
| **Soluble ions (mg/100 g dry weight soil)** | | | | | | | |  |  |
| **P** | | **Cl^−^** | **CO_3_^2-^** | **HCO^3−^** | **SO4^2-^** | **Mg^2+^** | **Na^+^** | **K^+^** |  |
| 1.28 | | 1.67 | - | 0.49 | 1.45 | 0.53 | 0.98 | 0.38 |  |

**Supplementary table 3** Effect of seed priming with MLE on the investigated parameters of two rice varieties Giza 177 and SG 65 grown under increased levels of seawater at vegetative stage.

|  | Dependent variables | Main variables and interactions | | | | | | |
| --- | --- | --- | --- | --- | --- | --- | --- | --- |
|  |  | Varieties (V) | Moringa Leaf Extract (MLE) | Salinity (S) | V × MLE | V × S | MLE × S | V × MLE × S |
|  | *Df* | 1 | 1 | 2 | 1 | 2 | 2 | 2 |
| Na^+^ and K^+^ content in shoot and root | Shoot Na^+^ content  Root Na^+^ content  Shoot K^+^ content  Root K^+^ content  Shoot K^+^/Na^+^ ratio  Root K^+^/Na^+^ ratio  Shoot Na^+^ content | **  **  ns  ns  **  **  ** | ***  ***  ***  ***  ***  ***  *** | ***  ***  ***  ***  ***  ***  *** | ***  ns  *  ns  ns  ns  *** | ns  ns  ns  ns  ns  ns  ns | ns  ns  ns  ns  **  ns  ns | ns  ns  ns  ns  ns  ns  ns |
| Photosynthetic pigments | Chl a  Chl b  Chl a+b  Total pigments  Carotenoids | ***  ***  ***  ***  *** | ***  ***  ***  ***  *** | ***  ***  ***  ***  *** | ***  **  ***  ***  *** | ***  ***  ***  ***  *** | ***  ***  ***  ***  *** | ***  *  ***  ***  *** |
| Gas exchange characteristics | *P_N_*  *E*  *gs*  *Ci*  WUE | ***  **  ***  ***  *** | ***  ***  ***  ***  *** | ***  ***  ***  ***  *** | ***  ns  **  **  *** | ns  ns  ns  ns  ns | ns  ns  ns  ns  ns | ns  ns  ns  ns  ns |
| Osmolytes | Sucrose  TSS  TC  TSP  AsA  Proline | ***  ns  ***  ***  ***  *** | ***  ***  ***  ***  ***  *** | ***  ***  ***  ***  ***  *** | ***  ***  ***  ***  ***  *** | ***  ***  ***  ns  ***  ns | ***  *  ***  ns  ***  *** | ***  ***  ***  *  ***  ** |
| Oxidative stress markers | EC  MDA  H_2_O_2_ | ***  ***  *** | ***  ***  *** | ***  ***  *** | ***  ns  *** | ***  ns  *** | ***  ***  *** | ***  **  *** |
| Antioxidants | APX  PPO  CAT  POD  Phenols  Flavonoids | ***  ***  ***  ***  ***  *** | ***  ***  ***  ***  ***  *** | ***  ***  ***  ***  ***  *** | ***  ns  ***  ***  ***  ns | ns  ns  ns  ***  ns  * | *  ***  ***  ***  ***  ** | ns  ns  ***  ***  ns  *** |

V; varieties, S; salinity, and MLE; moringa leaf extract. *, **, and *** indicate significant differences at *p* ≤ 0.05, 0.01, and 0.001 and ns indicates non-significant differences. *Df;* degree of freedom.
